# Association Between Granular Changes on Epipharyngeal Endoscopy and Allergen Sensitization in Chronic Epipharyngitis

**DOI:** 10.3390/reports9030300

**Published:** 2026-09-06

**Authors:** Manabu Mogitate

**Affiliations:** Mogitate ENT Clinic, Kawasaki 213-0011, Kanagawa, Japan; mogitateentjp@m08.itscom.net

**Keywords:** chronic nasopharyngeal inflammation, chronic epipharyngitis, granular changes, allergen sensitization, mite-specific IgE, house dust-specific IgE, nasopharyngeal endoscopy

## Abstract

**Background:** Granular changes observed under band-limited epipharyngeal endoscopy are considered a characteristic finding of chronic epipharyngitis; however, their biological significance remains unclear. This study investigated whether granular changes are associated with allergen sensitization and peripheral allergic biomarkers in patients with chronic epipharyngitis. **Methods:** A retrospective analysis was conducted in 59 patients with chronic epipharyngitis. Granular changes were graded endoscopically as 0 (absent), 1 (mild-to-moderate), or 2 (severe). Nasal eosinophil counts, peripheral blood eosinophil counts, total immunoglobulin E (IgE), mite-specific IgE, house dust-specific IgE, and peripheral blood CD4/CD8 ratios were evaluated. Comparisons among groups were performed using the Kruskal–Wallis and Mann–Whitney U tests, and correlations were assessed using Spearman’s rank correlation analysis. **Results:** Patients with granular changes demonstrated significantly higher mite-specific IgE and house dust-specific IgE levels than those without granular changes. In comparisons between patients without granular changes (grade 0) and those with granular changes (grades 1 and 2), significant differences were observed for mite-specific IgE (*p* = 0.016) and house dust-specific IgE (*p* = 0.016). In contrast, nasal eosinophil counts, peripheral blood eosinophil counts, total IgE levels, and CD4/CD8 ratios showed no significant associations with granular changes. Furthermore, Spearman correlation analysis revealed no significant correlations between granular change scores and any evaluated biomarker. **Conclusions:** Granular changes on epipharyngeal endoscopy were associated with sensitization to mite and house dust allergens but were not associated with peripheral eosinophil counts, total IgE levels, or CD4/CD8 ratios. These findings suggest that the presence of granular changes may be associated with allergen sensitization; however, no association with increasing granular severity was observed. Further studies incorporating local immune profiling and cytokine analyses are warranted to clarify the biological significance of granular changes.

## 1. Introduction

Chronic epipharyngitis is a persistent inflammatory disorder of the epipharyngeal mucosa that is associated with symptoms such as postnasal drip, throat discomfort, chronic cough, and sputum production [1,2,3]. The epipharynx is an immunologically active component of nasopharynx-associated lymphoid tissue (NALT), containing abundant lymphocytes, dendritic cells, and antigen-presenting cells involved in both innate and adaptive immune responses [4]. In Japan, chronic epipharyngitis is primarily diagnosed based on endoscopic findings and contact bleeding during epipharyngeal abrasive therapy (EAT), although objective biomarkers reflecting disease phenotypes remain limited [1,2,3]. Increasing evidence suggests that chronic epipharyngitis may be associated not only with local symptoms but also with systemic disorders, including immunoglobulin A (IgA) nephropathy, chronic fatigue syndrome, and post-coronavirus disease conditions through persistent mucosal immune activation [1,2,3,4].

The epipharynx represents an immunologically active mucosal organ that forms part of Waldeyer’s ring and contains abundant lymphoid tissue involved in antigen presentation and mucosal immune responses [4,5]. Epipharyngeal lymphoid tissue contains abundant B lymphocytes, CD4-positive T cells, dendritic cells, and macrophages and functions as an important interface between innate and adaptive immunity [4,5]. Recent molecular studies demonstrated increased expression of pro-inflammatory cytokines, including interleukin-6 (IL-6) and tumor necrosis factor-α (TNF-α), within the epipharyngeal mucosa of patients with chronic epipharyngitis, and these inflammatory signals were reduced following EAT [6]. Furthermore, increased numbers of epipharyngeal CD4-positive T cells have been observed in patients with chronic epipharyngitis and repeated EAT reduced these cell populations in parallel with symptomatic improvement [7]. These findings support the concept that chronic epipharyngitis is not merely a local structural abnormality but a disease characterized by active mucosal immune dysregulation rather than solely a consequence of chronic mucosal irritation.

Chronic epipharyngitis has increasingly been recognized as a condition that may influence systemic disorders through persistent activation of epipharyngeal lymphoid tissue. Previous studies have suggested associations with several systemic conditions, including immunoglobulin A (IgA) nephropathy, chronic fatigue syndrome, and post-viral conditions such as long COVID [8,9,10,11,12,13,14]. These observations support the concept that the epipharynx functions as an immunologically active mucosal site; however, objective biomarkers reflecting disease phenotypes remain limited.

Epipharyngeal abrasive therapy (EAT), originally developed as a local treatment for chronic epipharyngitis, has been reported to exert beneficial effects beyond local symptom improvement through modulation of epipharyngeal mucosal immunity. These observations further support the potential systemic relevance of epipharyngeal inflammation [6,7,12,13,14].

Clinically, granular changes, a characteristic endoscopic finding observed under band-limited light endoscopy [15], are often observed in patients who also exhibit findings suggestive of allergic rhinitis, such as pale inferior turbinates. This observation raises the possibility that granular changes may be associated with allergic sensitization or allergen-driven mucosal inflammation. However, whether granular changes are associated with established allergic biomarkers has not been systematically investigated.

Previous studies evaluating chronic nasopharyngeal inflammation have primarily focused on peripheral eosinophil counts, serum immunoglobulin E (IgE), exhaled nitric oxide, and other systemic immune markers [16,17]. Nevertheless, local mucosal immune responses do not necessarily correspond to peripheral blood biomarkers, and allergic sensitization may exist even in the absence of marked eosinophilia or elevated total IgE levels. Therefore, the relationship between granular changes and allergen-specific sensitization remains uncertain.

The present study aimed to investigate the association between granular changes observed on epipharyngeal endoscopy and allergen sensitization in patients with chronic epipharyngitis. Specifically, we evaluated the relationships between granular changes and mite-specific IgE, house dust-specific IgE, peripheral eosinophil counts, total IgE levels, and CD4/CD8 ratios to clarify the immunological significance of this characteristic endoscopic finding.

## 2. Materials and Methods

### 2.1. Study Design and Participants

This retrospective observational study was conducted as a post hoc sub-analysis of a previously published study investigating CD4/CD8 profiles in epipharyngeal abrasive cells in patients with chronic epipharyngitis.

The original study included patients with chronic epipharyngitis who underwent immunological evaluation at Mogitate ENT Clinic between June 2021 and September 2021. Chronic nasopharyngeal inflammation was diagnosed based on characteristic endoscopic findings and the presence of contact bleeding during EAT, which is a commonly used diagnostic approach in Japan. Because diagnostic criteria for chronic epipharyngitis have not been standardized internationally, caution is required when generalizing these findings to other clinical settings.

Patients with available endoscopic images and allergic biomarker data at the initial visit were included in the present analysis. Only patients with evaluable endoscopic images and complete allergic biomarker data available at the initial visit were included in the final analysis. Patients with missing endoscopic images or incomplete biomarker data were excluded.

### 2.2. Ethical Approval

This study was conducted in accordance with the principles of the Declaration of Helsinki and was approved by the Ethics Review Committee of Ota General Hospital, Kawasaki, Japan (approval number 22026; approved on 17 September 2022).

The present study represents a post hoc sub-analysis of a previously published study evaluating CD4/CD8 profiles in patients with chronic epipharyngitis.

### 2.3. Endoscopic Evaluation

Endoscopic examination was performed using a flexible transnasal endoscope (HOYA Corporation, Tokyo, Japan; EPK-i7000 processor; VNL-1190STK scope). Conventional white-light imaging was used to evaluate mucosal redness, swelling, mucus/crust adhesion, and contact bleeding. In addition, band-limited light imaging was used to assess granular changes.

Granular changes were graded as follows:

- 0 = absent
- 1 = mild to moderate
- 2 = severe

Patients were classified into three groups according to the severity of granular changes. All endoscopic examinations and laboratory assessments were performed at the initial visit before initiation of Epipharyngeal Abrasive Therapy (EAT). Endoscopic grading was performed before laboratory results became available.

### 2.4. Allergic Biomarkers

The following allergic and immunological biomarkers were evaluated:

- Nasal eosinophil count
- Peripheral blood eosinophil count
- Total serum immunoglobulin E (IgE)
- Mite-specific IgE
- House dust-specific IgE
- Peripheral blood CD4/CD8 ratio

Nasal eosinophils were assessed using routine nasal smear examination performed at the initial visit. Mite-specific IgE and house dust-specific IgE were interpreted as markers of allergen sensitization and were measured using the ImmunoCAP fluorescence enzyme immunoassay (CAP-FEIA) method. Peripheral blood eosinophil counts, serum IgE concentrations, allergen-specific IgE levels, and lymphocyte subset analyses were obtained from routine clinical laboratory testing performed at the initial visit.

Mite-specific IgE and house dust-specific IgE were used as indicators of allergen sensitization. Serum allergen-specific IgE levels were measured by a commercial laboratory (SRL, Tokyo, Japan) using the ImmunoCAP fluorescence enzyme immunoassay (CAP-FEIA) method and were expressed in UA/mL. Values reported as 0.00 were considered to be below the lower limit of detection of the assay.

### 2.5. Statistical Analysis

Continuous variables were expressed as median values and interquartile ranges (IQRs).

The primary analysis compared patients without granular changes (Grade 0) and those with granular changes (Grades 1 and 2 combined). Comparisons among the three granular change groups (grades 0, 1, and 2) and correlation analyses were conducted as secondary exploratory analyses. Comparisons among the three granular change groups were performed using the Kruskal–Wallis test. When a significant overall difference was identified, post-hoc pairwise comparisons were performed using Dunn’s test with Bonferroni correction.

To evaluate the association between the presence of granular changes and allergen sensitization, patients were additionally categorized into granular change absent (Grade 0) and granular change present (Grades 1 and 2 combined). Grades 1 and 2 were combined because the study focused on the presence versus absence of granular changes, and the small number of Grade 2 patients (*n* = 5) limited the statistical power of severity-based analyses. The two groups were compared using the Mann–Whitney U test. An exploratory comparison was also performed between severe granular changes (Grade 2) and non-severe granular changes (Grades 0 and 1) using the Mann–Whitney U test.

Associations between granular change scores and allergic biomarkers were evaluated using Spearman’s rank correlation coefficient.

All statistical tests were two-sided, and a *p* value < 0.05 was considered statistically significant. Given the exploratory post hoc nature of the study, *p*-values should be interpreted cautiously because multiple statistical comparisons were performed. Statistical analyses were performed using Python 3.12 (NumPy, pandas, and SciPy libraries).

## 3. Results

### 3.1. Patient Characteristics

A total of 59 patients were included in the analysis. The median age was 50 years (IQR, 41.5–60.0 years), and 14 patients (23.7%) were male. Patient characteristics are summarized in Table 1.

**Figure 1:**
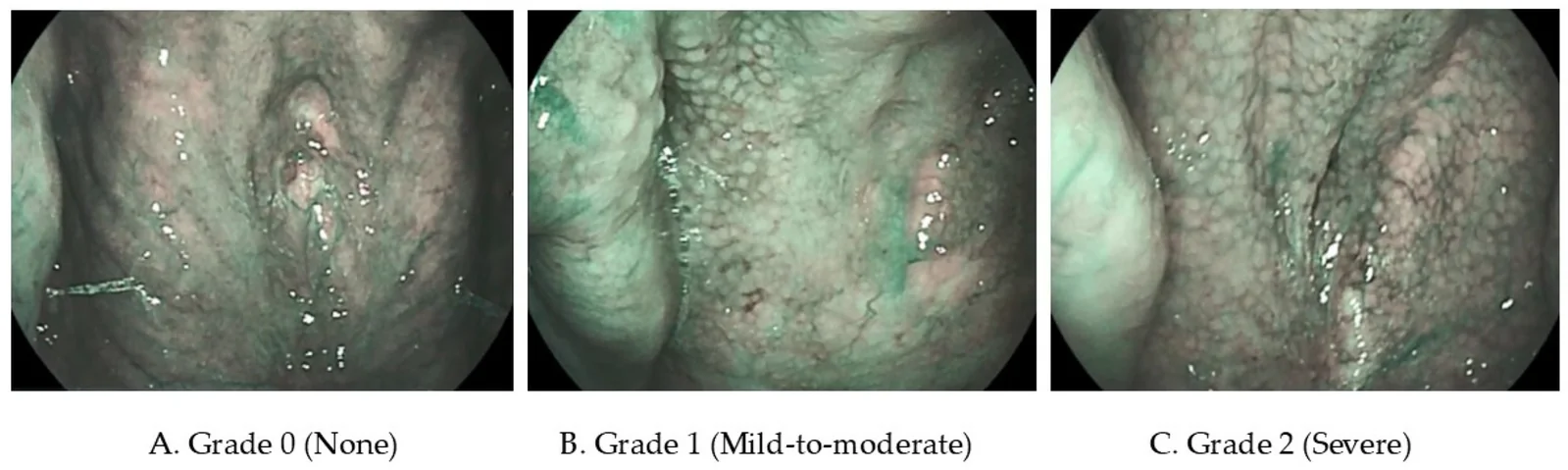
Representative Grades of Granular Changes Observed Under Band-Limited Endoscopy. Representative band-limited endoscopic images demonstrating the grading of granular changes. Grade 0 indicates the absence of granular changes, Grade 1 represents mild-to-moderate cobblestone-like mucosal alterations, and Grade 2 represents severe granular changes with prominent granular mucosal irregularity. Granular changes were evaluated according to the grading system described in the Section 2.

According to the severity of granular changes, 13 patients (22.0%) were classified as grade 0, 41 (69.5%) as grade 1, and 5 (8.5%) as grade 2. Representative endoscopic images of granular changes are shown in Figure 1.

### 3.2. Comparison Among the Three Granular Change Groups

Comparisons of allergic biomarkers among patients with grade 0, grade 1, and grade 2 granular changes are summarized in Table 2.

### 3.3. Comparison Between Patients with and Without Granular Changes

Baseline demographic characteristics of patients without granular changes (Grade 0) and those with granular changes (Grades 1 and 2 combined) are summarized in Table 3.

Comparisons between patients without granular changes (grade 0) and those with granular changes (grades 1 and 2) are summarized in Table 4.

No significant differences were observed for nasal eosinophil counts, peripheral blood eosinophil counts, total serum IgE levels, or CD4/CD8 ratios.

**Figure 2:**
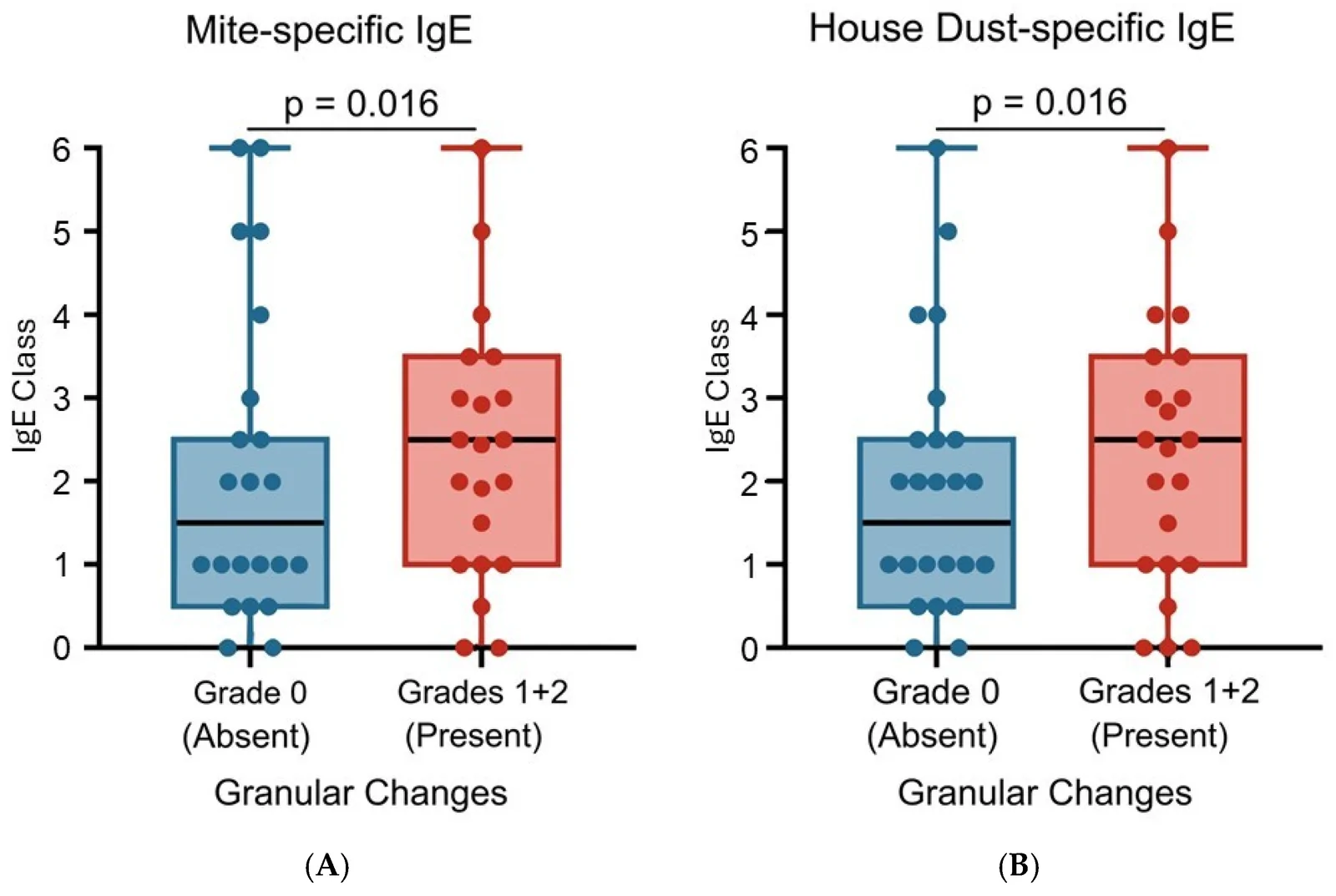
Associations between granular changes and allergen-specific IgE levels. (**A**) Comparison of mite-specific immunoglobulin E (IgE) levels between patients without granular changes (Grade 0) and those with granular changes (Grades 1 and 2). (**B**) Comparison of house dust-specific IgE levels between patients without granular changes (Grade 0) and those with granular changes (Grades 1 and 2). Patients with granular changes demonstrated significantly higher mite-specific IgE and house dust-specific IgE levels than patients without granular changes (both *p* = 0.016, Mann–Whitney U test). The boxes represent the interquartile range (IQR), the horizontal line indicates the median value, and whiskers indicate the data range.

The distributions of mite-specific IgE and house dust-specific IgE levels are shown in Figure 2.

### 3.4. Comparison Between Severe and Non-Severe Granular Change

Patients with severe granular changes (grade 2) were compared with those without severe granular changes (grades 0 and 1).

No significant differences were detected in any evaluated allergic biomarker, including nasal eosinophil counts, peripheral blood eosinophil counts, total serum IgE levels, allergen-specific IgE levels, or CD4/CD8 ratios (all *p* > 0.05).

Therefore, increasing endoscopic severity of granular changes was not accompanied by elevations in systemic allergic biomarkers.

### 3.5. Correlation Between Granular Change Scores and Allergic Biomarkers

Spearman correlation analyses are summarized in Table 5.

No significant correlations were identified between granular change scores and any evaluated biomarker, including mite-specific IgE and house dust-specific IgE.

## 4. Discussion

The present study investigated the relationship between epipharyngeal granular changes observed under band-limited endoscopy and allergic biomarkers in patients with chronic epipharyngitis. The principal finding was that the presence of granular changes was associated with significantly higher mite-specific IgE and house dust-specific IgE levels. While significant associations were observed for the presence of granular changes, increasing endoscopic severity was not accompanied by progressively higher specific IgE levels. In contrast, no significant associations were observed for nasal eosinophil counts, peripheral blood eosinophil counts, total IgE levels, or peripheral blood CD4/CD8 ratios. Furthermore, granular change scores were not significantly correlated with any evaluated biomarker. These findings suggest an association between epipharyngeal granular changes and allergen sensitization. However, because local inflammatory markers were not directly assessed, the present findings do not demonstrate local allergic inflammation within the epipharyngeal mucosa. Given that mite and house dust allergens represent major perennial inhalant antigens, the results raise the possibility that granular changes may be associated with allergen sensitization in patients with chronic epipharyngitis.

Granular changes are a characteristic endoscopic finding frequently observed in patients with chronic epipharyngitis and are readily visualized using band-limited endoscopy [2,18,19]. Although these cobblestone-like mucosal alterations have long been recognized in daily clinical practice, their biological significance has remained poorly understood [18,19]. Patients with prominent granular changes frequently exhibit concomitant findings suggestive of allergic rhinitis, including pale inferior turbinates, raising the possibility of an association between granular changes and allergic airway inflammation. The present results provide objective support for this clinical observation by demonstrating significant associations between the presence of granular changes and sensitization to mite and house dust allergens.

The epipharyngeal mucosa is lined by ciliated respiratory epithelium and is continuously exposed to inhaled environmental antigens. Similar to other respiratory mucosal surfaces, repeated exposure to perennial allergens may induce local immune activation within the epipharyngeal mucosa and nasopharynx-associated lymphoid tissue [20,21]. Although Th2-related cytokines such as interleukin-4, interleukin-5, and interleukin-13 were not directly evaluated in the present study, the association between granular changes and perennial allergen sensitization suggests a possible relationship between these endoscopic findings and allergen sensitization.

Interestingly, mite-specific and house dust-specific IgE levels were significantly associated with the presence of granular changes, whereas total IgE levels were not. However, increasing granular severity was not accompanied by progressively higher allergen-specific IgE levels, and no clear dose–response relationship was observed. Similar discrepancies have been described in allergic airway diseases, where allergen-specific sensitization may be present despite relatively low total IgE levels and limited peripheral eosinophilia [21,22,23,24]. These observations suggest that allergen-specific immune responses may provide more relevant information than conventional systemic biomarkers when evaluating mucosal inflammatory phenotypes.

In the present study, sensitization to perennial allergens appeared to be more closely related to the presence of granular changes than markers reflecting overall systemic allergic status [21,22,23,24]. This finding may indicate that chronic exposure to specific inhalant allergens contributes to localized inflammatory responses within the epipharyngeal mucosa. Accordingly, evaluation of allergen-specific sensitization may offer greater insight into the biological background of granular changes than measurements of total IgE alone.

Another important observation was the absence of significant associations between granular changes and peripheral eosinophil counts. Eosinophils are generally regarded as major effector cells in type 2 inflammation; however, peripheral blood eosinophilia does not always reflect local mucosal immune activity [23,24]. Previous studies have demonstrated that allergic inflammation may occur within the airway mucosa despite normal peripheral eosinophil counts, supporting the concept of compartmentalized immune responses [23,24]. Therefore, the lack of a relationship between granular changes and peripheral eosinophils does not exclude the possibility that local type 2 inflammatory responses contribute to the development of granular changes.

This interpretation is supported by our recent investigation of the Th17/Treg axis in chronic epipharyngitis, in which mucosal immune profiles differed substantially from those observed in peripheral blood. In that study, mucosal Th17/Treg ratios were associated with endoscopic severity, whereas peripheral immune markers showed little relationship with local inflammatory findings [25]. Taken together, these observations suggest that immunological processes within the epipharyngeal mucosa may not be adequately reflected by conventional systemic biomarkers. A similar local–systemic dissociation may therefore exist for type 2 inflammatory responses, potentially explaining why granular changes were associated with allergen sensitization but not with peripheral eosinophil counts or total IgE levels.

The present findings further support the concept that local mucosal immunity and systemic immunological biomarkers may be dissociated in chronic epipharyngitis. The epipharynx contains abundant lymphoid tissue and forms part of nasopharynx-associated lymphoid tissue (NALT) and serves as an important site for antigen sampling, immune surveillance, and mucosal immune regulation [20,21]. Continuous exposure to inhaled environmental antigens may therefore generate localized immune responses that are only partially reflected in peripheral blood measurements. Previous studies have demonstrated increased expression of pre-inflammatory cytokines within the epipharyngeal mucosa of patients with chronic epipharyngitis [6]. Furthermore, we previously reported that repeated EAT reduced local CD4-positive cell infiltration in parallel with symptomatic improvement [7]. Taken together, these findings suggest that endoscopic findings may reflect biological processes occurring primarily within the epipharyngeal mucosal environment rather than systemic immune status alone.

Our findings should also be interpreted in light of previous observations regarding nitric oxide. We previously demonstrated that patients with granular changes exhibited significantly higher exhaled nitric oxide levels and that exhaled nitric oxide levels correlated with endoscopic severity scores [15,16]. Because nitric oxide is produced primarily by respiratory epithelial cells and is closely associated with airway inflammatory activity, those findings suggested that granular changes reflect biologically active epithelial inflammation rather than a simple structural mucosal alteration [26,27].

The present results provide additional context for interpreting those observations. Granular changes were associated with sensitization to mite and house dust allergens, both of which are major perennial inhalant allergens [21,22]. Although direct assessment of Th2-related cytokines was not performed, it is plausible that allergen-driven local immune activation contributes to both nitric oxide production and the development of granular mucosal changes [23,24,27]. Taken together, these findings suggest a possible association between granular changes and allergen sensitization; however, this hypothesis requires confirmation through direct local immune assessments.

Notably, this interpretation remains compatible with the absence of significant associations with peripheral eosinophil counts and total IgE levels. Similar to our recent findings regarding the Th17/Treg axis, local inflammatory processes within the epipharyngeal mucosa may not be adequately reflected by conventional systemic biomarkers, highlighting the importance of direct evaluation of the local mucosal environment [25].

Several limitations should be acknowledged. 

First, this was a retrospective single-center study with a relatively small sample size, particularly in the grade 2 subgroup. The limited number of patients with severe granular changes (Grade 2, n = 5) may have reduced the ability to detect significant severity-related associations. However, this distribution is consistent with previous endoscopic studies of chronic epipharyngitis, in which mild-to-moderate granular changes were observed more frequently than severe lesions.

Second, although the present findings suggest a potential association between granular changes and allergen-responsive mucosal inflammation, local type 2 inflammatory markers such as IL-4, IL-5, IL-13, eosinophil cationic protein, and tissue eosinophil infiltration were not evaluated. Therefore, a direct mechanistic relationship between granular changes and type 2 inflammation or local allergic inflammation could not be established.

Third, allergen sensitization was assessed using serum allergen-specific IgE levels, and direct analyses of local epipharyngeal immune responses were not performed. Recent studies have suggested that local and systemic immune responses may differ substantially in chronic epipharyngitis. Therefore, the immunological mechanisms underlying granular changes remain uncertain.

Fourth, multiple biomarkers were evaluated in this exploratory analysis, which may have increased the risk of type I error. Therefore, the present findings should be interpreted as hypothesis-generating and require confirmation in larger prospective studies. Future studies integrating endoscopic phenotyping, local immune profiling, cytokine analyses, and nitric oxide measurements will be necessary to clarify the relationship between granular changes and allergen sensitization in chronic epipharyngitis.

Fifth, formal interobserver and intraobserver reproducibility assessments were not performed. Therefore, the reproducibility of the endoscopic grading system should be evaluated in future studies.

Sixth, potential confounding factors, including allergic comorbidities, smoking status, medication use, seasonal variation, and previous treatment history, were not consistently available and therefore could not be adjusted for in the present analysis.

Seventh, no healthy control group was included in this study. Therefore, the present findings should be interpreted as associations observed within patients with chronic epipharyngitis rather than evidence that allergen sensitization plays a specific causal role in the disease.

Eighth, the diagnosis of chronic epipharyngitis was based on a diagnostic framework commonly used in Japan, incorporating characteristic endoscopic findings and contact bleeding during EAT. Therefore, the external validity of the present findings may be limited in regions where this diagnostic approach is not routinely applied.

**Figure 3:**
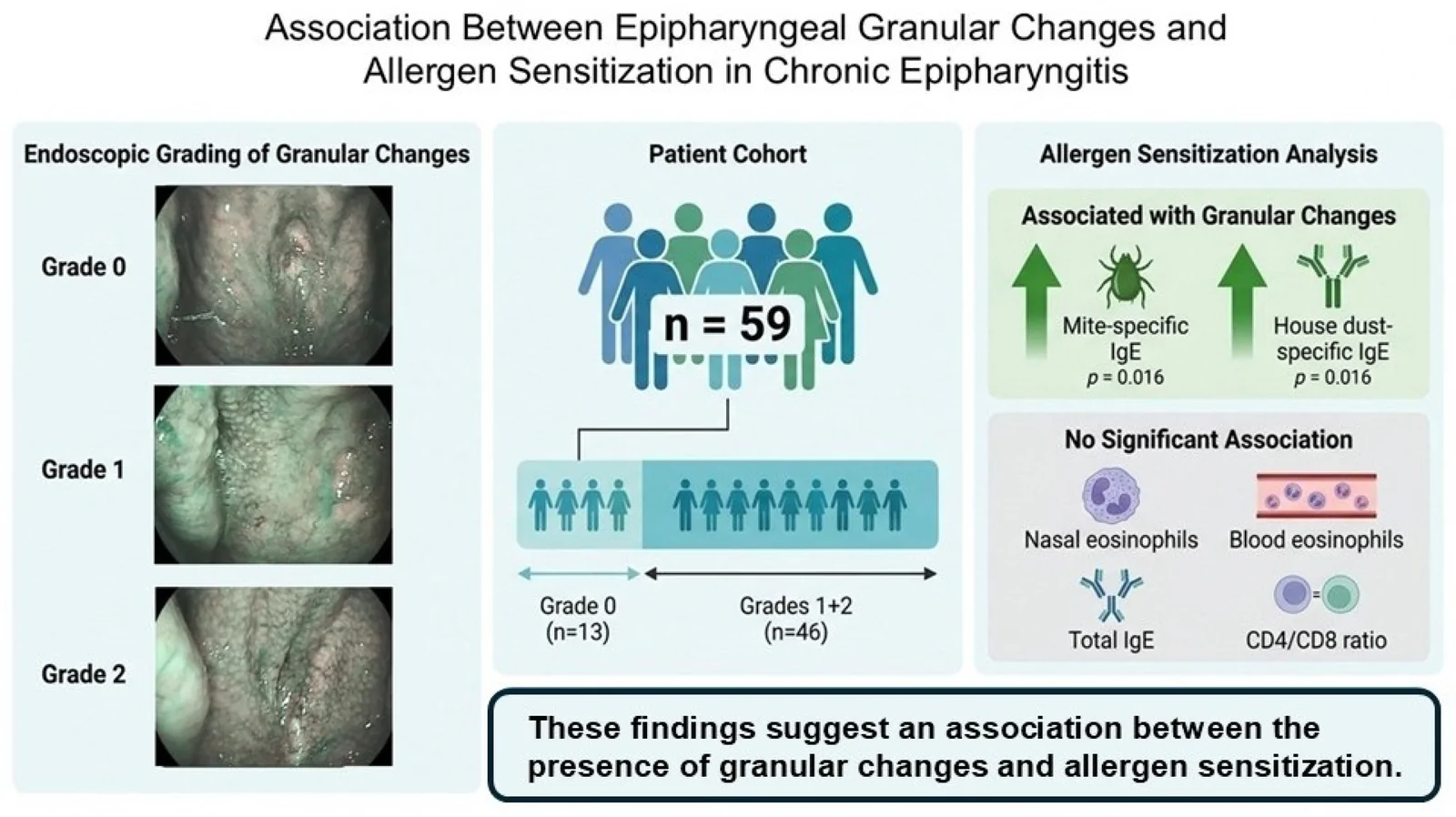
Graphical summary of the association between epipharyngeal granular changes and allergen sensitization in chronic epipharyngitis. Patients with epipharyngeal granular changes demonstrated significantly higher mite-specific IgE and house dust-specific IgE levels than patients without granular changes. In contrast, nasal eosinophil counts, peripheral blood eosinophil counts, total IgE levels, and CD4/CD8 ratios showed no significant associations. These findings suggest an association between the presence of granular changes and allergen sensitization. Arrows indicate the proposed relationship among granular changes, allergen sensitization, and chronic epipharyngitis.

A graphical summary of the present findings and the proposed hypothesis is presented in Figure 3.

Despite these limitations, this study provides the first evidence that epipharyngeal granular changes are associated with sensitization to mite and house dust allergens. The absence of significant relationships with peripheral eosinophil counts, total IgE levels, and CD4/CD8 ratios suggests that local mucosal immune processes may be more relevant than conventional systemic biomarkers in the development of granular changes. Together with previous observations linking granular changes to elevated exhaled nitric oxide levels, the present findings suggest a possible association between granular changes and allergen sensitization. Further studies using direct assessment of local mucosal immune responses are needed to clarify the underlying biological mechanisms. Further prospective studies incorporating local cellular analyses, cytokine profiling, nitric oxide measurements, and immunophenotyping are warranted to clarify the biological significance and immunological mechanisms underlying granular changes.

## Figures and Tables

**Table 1:** Baseline Characteristics of the Study Population.

Variable	Value
Number of patients, n	59
Age, years, median (IQR)	50 (41.5–60.0)
Male sex, n (%)	14 (23.7)
Female sex, n (%)	45 (76.3)
Grade 0 (Absent), n (%)	13 (22.0)
Grade 1 (Mild-to-moderate), n (%)	41 (69.5)
Grade 2 (Severe), n (%)	5 (8.5)

**Table 2:** Comparisons of Allergic Biomarkers Among Granular Change Grades.

Variable	Grade 0	Grade 1	Grade 2	*p*
Nasal eosinophils	0.00 (0.00–1.00)	0.00 (0.00–1.00)	0.50 (0.00–1.00)	0.634
Blood eosinophils	101.20 (61.20–258.40)	166.20 (100.30–270.95)	143.15 (118.53–262.88)	0.484
Total IgE	34.20 (16.70–68.00)	50.95 (29.67–160.75)	154.00 (44.50–252.25)	0.371
Mite-specific IgE	0.00 (0.00–0.00)	1.50 (0.00–3.00)	0.00 (0.00–0.75)	0.03
House dust-specific IgE	0.00 (0.00–0.00)	0.50 (0.00–2.00)	0.00 (0.00–0.75)	0.039
CD4/CD8 ratio	2.79 (1.88–4.82)	2.61 (1.32–3.15)	2.86 (1.92–4.47)	0.392

Significant differences were observed for mite-specific IgE (*p* = 0.030) and house dust-specific IgE (*p* = 0.039). No significant differences were observed for nasal eosinophil counts, peripheral blood eosinophil counts, total serum IgE levels, or CD4/CD8 ratios.

**Table 3:** Baseline Demographic Characteristics of Patients With and Without Granular Changes.

Variable	Grade 0 (n = 13)	Grades 1 + 2 (n = 46)	*p* Value
Age, median (IQR)	52 (49–65)	48 (40.3–60)	0.176
Male sex, n (%)	4 (30.8%)	10 (21.7%)	0.485

**Table 4:** Comparison of Allergic Biomarkers Between Patients With and Without Granular Changes.

Variable	Grade 0 (n = 13)	Grades 1 + 2 (n = 46)	*p* Value
Nasal eosinophils	0.00 (0.00–1.00)	0.00 (0.00–1.00)	0.387
Blood eosinophils	101.20 (61.20–258.40)	149.60 (107.92–272.85)	0.242
Total IgE	34.20 (16.70–68.00)	53.95 (25.23–175.00)	0.21
Mite-specific IgE	0.00 (0.00–0.00)	1.00 (0.00–3.00)	0.016
House dust-specific IgE	0.00 (0.00–0.00)	0.00 (0.00–2.00)	0.016
CD4/CD8 ratio	2.79 (1.88–4.82)	2.65 (1.38–3.20)	0.261

Patients with granular changes demonstrated significantly higher mite-specific IgE levels (*p* = 0.016) and house dust-specific IgE levels (*p* = 0.016) than patients without granular changes.

**Table 5:** Spearman Correlations Between Granular Change Scores and Allergic Biomarkers.

Variable	Spearman ρ	*p* Value
Nasal eosinophils	0.125	0.347
Blood eosinophils	0.151	0.254
Total IgE	0.185	0.162
Mite-specific IgE	0.207	0.116
House dust-specific IgE	0.229	0.081
CD4/CD8 ratio	−0.081	0.543

## Data Availability

The clinical data used in this study cannot be shared publicly due to ethical restrictions and patient privacy protection. Access to the data is limited by the regulations of the institutional ethics committee, and the data are available from the corresponding author upon reasonable request.
